# Academy of Medicine Notes

**Published:** 1894-06

**Authors:** 


### Academy of Medicine Notes.

THE election of officers occurs Tuesday, June 26th, 4 P. M. to 6 P. M., in the Academy rooms. The only ticket put in nomination is the following: For president, P. W. Van Peyma, M. D.; for secretary, A. L. Benedict, M. D.; for treasurer, E. A. Smith, M. D.; for trustee, H. R. Hopkins, M. D.

THE annual meeting of the Academy will be held Tuesday, June 26, 1894. Mr. George W. Rafter, of Rochester, the distinguished sanitarian, will deliver an address on Intermittent Filtration in its Application to Domestic Filters.

THE meetings, sections and general, for June are: Surgery, June 5th; medicine, June 12th; stated meeting of the Academy, June 19th; annual meeting of the Academy, June 26th.

THE next stated meeting of the Academy occurs Tuesday, June 26th, under the auspices of the section on anatomy, physiology and pathology.

THE section on general medicine elected C. C. Wyckoff, M. D., president, and D. H. Sherman, M. D., secretary, at its last meeting.

